# Z3495, a LysR-Type Transcriptional Regulator Encoded in O Island 97, Regulates Virulence Gene Expression in Enterohemorrhagic *Escherichia coli* O157:H7

**DOI:** 10.3390/microorganisms12010140

**Published:** 2024-01-10

**Authors:** Qian Wang, Yi Wei, Yu Huang, Jingliang Qin, Bin Liu, Ruiying Liu, Xintong Chen, Dan Li, Qiushi Wang, Xiaoya Li, Xinyuan Yang, Yuanke Li, Hao Sun

**Affiliations:** 1 The Key Laboratory of Molecular Microbiology and Technology, Ministry of Education, Nankai University, Tianjin 300457, China; qianwangwqaq@163.com (Q.W.); 2120221542@mail.nankai.edu.cn (Y.H.); lixiaoyafighting@hotmail.com (X.L.);; 2 Tianjin Key Laboratory of Microbial Functional Genomics, TEDA Institute of Biological Sciences and Biotechnology, Nankai University, Tianjin 300457, China; 3 Nankai International Advanced Research Institute, Shenzhen 518045, China; 4 State Key Laboratory of Medicinal Chemical Biology, Nankai University, Tianjin 300071, China

**Keywords:** Enterohemorrhagic *Escherichia coli*, O islands, transcriptional regulator, virulence

## Abstract

Enterohemorrhagic *Escherichia coli* (EHEC) is an important foodborne pathogen that infects humans by colonizing the large intestine. The genome of EHEC O157:H7 contains 177 unique O islands (OIs). Certain OIs significantly contribute to the heightened virulence and pathogenicity exhibited by EHEC O157:H7. However, the function of most OI genes remains unknown. We demonstrated here that EHEC O157:H7 adherence to and colonization of the mouse large intestine are both dependent on OI-97. *Z3495*, which is annotated as a LysR-type transcriptional regulator and encoded in OI-97, contributes to this phenotype. Z3495 activated the locus of enterocyte effacement (LEE) gene expression, promoting bacterial adherence. Deletion of *z3495* significantly decreased the transcription of *ler* and other LEE genes, the ability to adhere to the host cells, and colonization in the mouse large intestine. Furthermore, the ChIP-seq results confirmed that Z3495 can directly bind to the promoter region of *rcsF*, which is a well-known activator of Ler, and increase LEE gene expression. Finally, phylogenetic analysis revealed that Z3495 is a widespread transcriptional regulator in enterohemorrhagic and enteropathogenic *Escherichia coli*. As a result of this study, we have gained a deeper understanding of how bacteria control their virulence and provide another example of a laterally acquired regulator that regulates LEE gene expression in bacteria.

## 1. Introduction

Enterohemorrhagic *Escherichia coli* (EHEC) O157:H7 is the source of many diseases in individuals due to its specific colonization of the large intestine [1]. As a result of ingestion of contaminated food, EHEC infections may result in mild diarrhea, and hemorrhagic colitis (HC), but they can also be fatal, including hemolytic uremic syndrome (HUS) [2,3]. Developing attaching and effacing (A/E) lesions is necessary for the colonization of EHEC O157:H7. A/E lesions involve the loss of microvilli, rearranging the underlying cytoskeleton of the host, and producing a pedestal-like structure beneath the bacterium [4,5].

In EHEC O157:H7, the locus of enterocyte effacement (LEE) pathogenicity island confers the ability to form A/E lesions. The LEE comprises five major operons (LEE1-5) that encode a type three secretion system (T3SS) and effectors [6]. Genes ranging from LEE2 to LEE5 are directly activated by the first gene of the LEE1 operon, *ler*, which encodes the master transcriptional regulator Ler [6]. The remaining genes in LEE1-3 encode the major structural components of T3SS [7]. LEE4 encodes the secreted proteins EspA, EspB, EspD, and EspF [8,9]. LEE5 encodes the major adhesin intimin Eae and its cognate ‘‘translocated intimin receptor’’ Tir, which are necessary for intimate adherence to host epithelial cells [10]. LEE transcriptional regulation is extremely complex, involving at least three regulator classes, namely, LEE-encoded, global, and horizontally transferred. The regulation works in either a Ler-dependent or a Ler-independent manner [11]. In Ler-dependent LEE regulators, LEE expression is controlled via Ler directly, whereas Ler-independent regulators directly regulate other LEE operons [12]. Although the A/E lesions’ formation is critical for EHEC virulence, the overall regulatory networks and the mechanisms through which the T3SS is activated to promote these virulence phenotypes are not fully understood.

During evolution, EHEC acquired ~1.34 Mb of unique DNA by lateral gene transfer, which contains 177 genomic islands, termed O islands (OIs) [13]. These OIs contain a number of virulence factors, effectors, and virulence-regulating proteins, as well as virulence-regulating sRNAs, contributing largely to the pathogenicity of EHEC O157:H7. Previous studies proved that eight OIs, including OI-15, OI-43, OI-45, OI-48, OI-57, OI-93, OI-122, and OI-148, encode virulence factors that serve to colonize and replicate within the host, neutralize defenses, and spread into new hosts [14,15]. The effectors encoded in OIs modulate diverse signaling pathways and physiological processes, such as ion secretion, apoptosis, membrane insertion, and cytoskeletal modification [15,16]. Virulence regulatory proteins and sRNAs play a vital role in regulating the expression of virulence genes, ensuring their expression only under optimal environmental conditions [17]. These islands encompass 1387 genes (26% of the total) and comprise the main known virulence elements in O157:H7, including LEE (OI-148) and Shiga toxin-converting phages (OI-45 and OI-93) [13,18]. Among these 1387 OI genes, 69 genes (4.97%) have been identified as being related to EHEC O157:H7 virulence, while the function of the remaining 1271 genes (91.64%) has been predicted or is unknown [15].

In this study, we found that deletion of OI-97 significantly reduced the ability of bacterial adherence to host cells, LEE gene expression, and colonization in the mouse intestine in O157. This phenotype is contributed by Z3495, a virulence regulatory protein encoded in OI-97. Δ*z3495* significantly reduced the ability to adhere to HeLa cells and the rate of colonization in the mouse intestine compared to the WT strain. By ChIP-seq analysis, we found that Z3495 binds to the promoter of *rcsF* directly, which induces the expression of LEE, contributing to virulence. This work reveals a new example of laterally acquired regulators for the control of EHEC O157:H7 virulence.

## 2. Materials and Methods

### 2.1. Bacterial Strains, Plasmids, and Cell Culture

The strains, plasmids, and oligonucleotides used in this study are listed in Supplementary Tables S1, S2 and S3, respectively. Mutant strains were obtained using the lambda red recombinase system [19]. Complementary strains were established by cloning *z3495* into the pBAD33 plasmid. Bacteria were grown overnight in Luria–Bertani (LB) broth with antibiotics when appropriate (ampicillin [100 μg/mL], chloramphenicol [50 μg/mL], and kanamycin [50 μg/mL]). 

### 2.2. Bacterial Adherence Assay

The methods for adherence assays have been described previously [20]. In brief, HeLa cells were seeded into 6-well plates with Dulbecco’s Modified Eagle Medium (DMEM) containing 10% FBS for at least 16 h. Then the cells were washed with sterile PBS and incubated with bacteria at a multiplicity of infection of 100 (OD_600_ = 0.4) in DMEM, then co-incubated for 3 h at 37 °C in an atmosphere with 5% CO_2_. After co-incubation, nonadherent bacteria were removed via extensive washing with sterile PBS three times. HeLa cells were then disrupted with 1 mL of 0.1% SDS for 5 min. The lysates were diluted and plated on LB agar. The attachment efficiency was determined by measuring the number of colonies per milliliter.

### 2.3. Quantitative Real-Time PCR (qRT-PCR)

The total RNA was extracted using a TRIzol reagent (Invitrogen, Waltham, MA, USA). First-strand cDNA synthesis was performed by a PrimeScript RT Reagent kit (Takara, San Jose, CA, USA). A qRT-PCR was performed using the SYBR Green PCR master mix on an ABI 7500 sequence detection system (Applied Biosystems, Foster City, CA, USA). Each Ct value was compared with the endogenous control 16S ribosomal RNA using the comparative Ct method. Fold differences were calculated based on ΔΔCt. All experiments were performed three times. 

### 2.4. Mouse Colonization Experiments

Six-week-old female BALB/c mice were allowed to acclimate for 7 days with ad libitum access to food and water. The mice were starved for 18 h before bacterial infection and orally infected by gavage with 100 mL of PBS containing 10^9^ CFUs of logarithmic-phase bacteria. The mice were anesthetized and euthanized by cervical dislocation after a 6 h infection. The colon was resected, and the luminal contents were then removed. The colon was thoroughly and carefully washed and weighed. Following homogenization in 0.5 mL of PBS, the homogenates were subsequently diluted and plated on LB agar. The adherence efficiency in vivo was determined by measuring the number of colonies per gram of colon.

### 2.5. Electrophoretic Mobility Shift Assays

EMSA assays were performed as previously reported. Briefly, the LEE promoters, *rcsF* promoters, and the *rpoS* fragments were amplified. The primers are shown in Supplementary Table S3. The gel was purified using a MinElute gel extraction kit (Qiagen, Germantown, MD, USA). *Z3495* was cloned into the pMal-c5X vector and purified in *E. coli* strain BL21 (DE3). Varying amounts of Z3495 at 0–4 μM were incubated with 1 μL of DNA probe (40 ng) in a 20 μL final volume for 20 min at room temperature. The binding buffer used consisted of 1 mM of Tris-HCl [pH 7.5], 0.2 mM of dithiothreitol, 5 mM of MgCl_2_, 10 mM of KCl, and 50% glycerol. The reaction mixtures were then subjected to electrophoresis on a 6% polyacrylamide gel in 0.5 × TBE buffer (44.5 mM Tris, 44.5 mM of boric acid, and 1 mM of EDTA, pH 8.0) at 4 °C and 80 V/cm for 90 min. The gel was stained with GelRed nucleic acid staining solution in 0.5 × TBE buffer for 10 min.

### 2.6. ChIP-seq Analysis

ChIP was performed as previously described with some modifications [21,22]. Inducible expression vectors carrying 3 × FLAG-tagged *z3495* were constructed and transformed into the EHEC O157:H7 Δ*z3495*. The bacterial cultures were grown to an OD_600_ of 0.4, and protein expression was then induced by incubation with 1 mM IPTG for 30 min at 37 °C. The cultures were harvested, crosslinked, sonicated, and immunoprecipitated with anti-3 × FLAG antibody (Sigma, Livonia, MI, USA; #F1804) and protein A magnetic beads (Invitrogen, Carlsbad, CA, USA, #10002D) to enrich the protein-DNA complexes. As a negative control, ChIP was performed on another aliquot without antibody addition. RNase A and proteinase K were used to remove RNA and protein. The DNA sample was purified using a PCR purification kit (Qiagen, Hilden, Germany). DNA fragments (150–250 bp) were selected for library construction. The libraries were sequenced using the HiSeq 2000 system (Illumina, San Diego, CA, USA). The reads were mapped to the *E. coli* O157:H7 EDL933 genomes using Burrows-Wheeler Aligner [23]. MACS software (version 2.2.7.1) was used to call peaks [24].

### 2.7. ChIP-qPCR

The relative enrichment of the promotors of *rcsF* and *rpoS* (the negative control) in the ChIP samples were examined with qRT-PCR using the SYBR Green PCR master mix. Relative enrichment was calculated using the formula 2^−ΔΔCt^, as previously described [25].

### 2.8. Statistical Analysis

All statistical analyses were performed using GraphPad Prism software v7.0. Data are shown as bar graphs or dot plots (mean ± SD). Statistical analyses were performed by two-tailed Student’s *t*-test, Mann-Whitney test, one-way ANOVA, or two-way ANOVA with Dunnett’s or Sidak’s post hoc test. A *p*-value < 0.05 was considered to indicate statistical significance.

## 3. Results

### 3.1. OI-97 Is Required for O157 Adherence and Colonization

**Figure 1:**
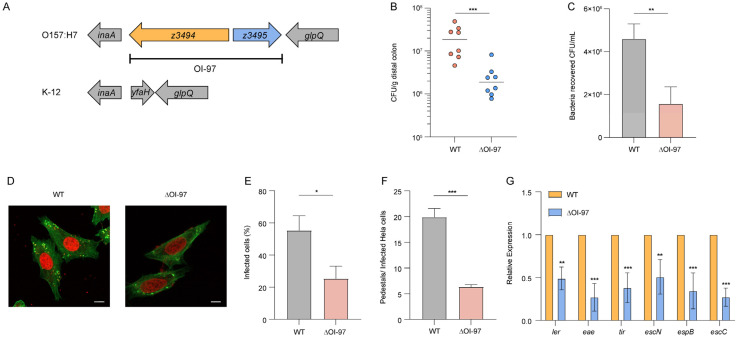
Deletion of OI-97 attenuates EHEC O157:H7 virulence. (**A**) Graphic representation of the region surrounding OI-97 in the genome of O157 EDL933 compared to *E. coli* K-12. Arrows represent open reading frames. (**B**) Evaluation of the adherence capacity of O157 WT and ΔOI-97 in the distal colon of mice at 6 h. (**C**) Adhesion of O157 WT and ΔOI-97 strains to HeLa cells in DMEM. (**D**) FAS of HeLa cells infected with WT and ΔOI-97. The actin cytoskeleton (green) and nuclei of the HeLa cell (red) are shown. Scale bar, 10 μm. (**E**) Quantification of the proportion of infected HeLa cells. (**F**) Quantification of the number of pedestals per infected HeLa cell. (**G**) qRT-PCR of the expression of LEE genes in WT and ΔOI-97. Data represent mean ± SD (n = 3). * *p* ≤ 0.05, ** *p* ≤ 0.01, and *** *p* ≤ 0.001 (Student’s *t*-test).

OI-97 in O157 is a 2006-bp region containing two open reading frames, *z3494* and *z3495*, with an unknown function (Figure 1A). To investigate whether OI-97 is associated with the virulence and pathogenicity of EHEC O157:H7, we first constructed the ΔOI-97 and determined its colonization capacity in the host intestinal tract. Six-week-old BALB/c mice were intragastrically inoculated with WT and ΔOI-97 strains, respectively. After 6 h of infection, the number of bacteria recovered from the colon homogenates was determined. The results showed that mice colons infected with ΔOI-97 had relatively fewer (9.87-fold) bacteria than the colons infected with the WT strain (Figure 1B). This result suggested that OI-97 exerted a positive effect on O157 virulence in vivo. 

We next investigated whether OI-97 affected EHEC O157:H7 adherence ability in host epithelial cells. The adherence assay results showed that the adherence ability of ΔOI-97 to HeLa cells was significantly lower (2.93-fold) than that of the WT (Figure 1C), indicating that OI-97 is required for O157 adherence in vitro. Growth curve analysis demonstrated that WT and ΔOI-97 shared similar rates in LB medium (Figure S1) both in the log phase and stationary phase, indicating that the positive effect of OI-97 on EHEC O157:H7 adherence was not due to faster growth rates. 

Since the most important features associated with O157 adhesion to host cells are the AE lesion and pedestal formation, a fluorescein actin staining (FAS) assay was performed to observe the presence of pedestals in HeLa cells infected by the WT or ΔOI-97. The results revealed that the percentage of AE lesion formation on HeLa cells in ΔOI-97 significantly decreased compared with that on the O157 WT (300 cells per strain, shown in Figure 1D,E). Meanwhile, the average pedestal number in ΔOI-97 infected cells decreased (6.35) compared with that of the O157 WT (19.9, Figure 1F). These results suggested that OI-97 promoted EHEC O157:H7 A/E lesion formation on host HeLa cells. Given that the AE lesion formation was mainly contributed by the LEE island, we further investigated the expression of six representative LEE genes. The qRT-PCR results showed that the expression of six representative LEE genes (*ler*, *eae*, *tir*, *espB*, *espC*, and *espN*) was significantly downregulated in ΔOI-97 (Figure 1G). These results indicated that OI-97 is required for O157 adherence to host cells and for LEE gene expression to promote its pathogenicity.

### 3.2. Z3495 Is Required for O157 Adherence and Colonization

**Figure 2:**
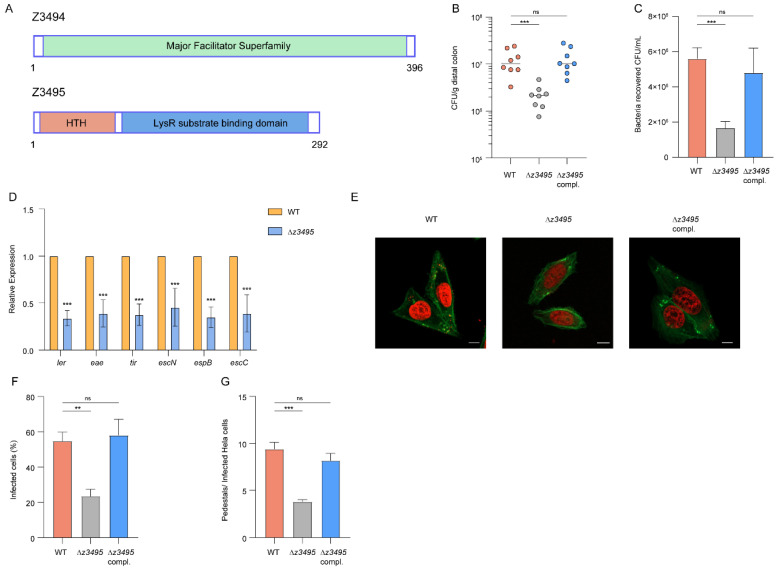
Deletion of *z3495* attenuates EHEC O157:H7 virulence. (**A**) Domain structure of Z3494 and Z3495. (**B**) Evaluation of the adherence capacity of the O157 WT, Δ*z3495*, and Δ*z3495* complementary strain in the distal colon of mice at 6 h. (**C**) Adhesion of the O157 WT, Δ*z3495*, and Δ*z3495* complementary strain to the HeLa cells in DMEM. (**D**) qRT-PCR of the expression of LEE genes in WT and Δ*z3495*. (**E**) FAS of HeLa cells infected with WT, Δ*z3495*, and Δ*z3495* complementary strains. The actin cytoskeleton (green) and nuclei of the HeLa cell (red) are shown. Scale bar, 10 μm. (**F**) Quantification of the proportion of infected HeLa cells. (**G**) Quantification of the number of pedestals per infected HeLa cell. Data represent mean ± SD (n = 3). ** *p* ≤ 0.01, and *** *p* ≤ 0.001 (Student’s *t*-test), ns, not significant.

The detailed domain structure analysis revealed that *z3494* contains an MFS_YfcJ superfamily motif, which is commonly found in major facilitator superfamily (MFS) transporters that function as moving substrates, including inorganic and organic ions, nucleosides, amino acids, short peptides, and lipids, across membranes (Figure 2A) [26]. Z3495 contains an N-terminal DNA-binding helix-turn-helix (HTH) motif, which is conserved in the LysR-type transcriptional regulator (LTTR) family (Figure 2A). To further elucidate which gene was responsible for the defective virulence phenotype observed in ΔOI-97, Δ*z3494,* and Δ*z3495* were constructed to determine the capacity of intestinal colonization in mice. The results showed that Δ*z3495* significantly reduced the number of bacteria recovered from the mouse colon compared with that of the WT and restored to the WT levels by plasmid complementation, while no significant difference was observed in Δ*z3494* (Figure 2B and Figure S2). These results revealed that *z3495* promotes intestinal colonization in O157 in vivo, contributing to virulence. To determine the capacity to adhere to host epithelial cells, we performed the bacterial adherence assay. The ability of Δ*z3495* to adhere to HeLa cells was significantly lower than that of the WT (3.36-fold) and was restored to the WT levels by plasmid complementation (Figure 2C), while Δ*z3494* did not obviously affect EHEC O157:H7 adherence (Figure S3). Notably, WT, Δ*z3495*, and Δ*z3495* complement strains grew at similar rates in vitro (Figure S4), indicating that the decreased colonization and cell adhesion of the Δ*z3495* was not due to slower bacterial growth. These results suggested that the attenuation of virulence in O157 ΔOI-97 depends on *z3495*. 

Furthermore, the expression of six representative LEE genes (*ler*, *eae*, *tir*, *espB*, *espC*, and *espN*) was significantly downregulated in Δ*z3495*, while no significant differences were observed in Δ*z3494* (Figure 2D and Figure S5). These results indicated that *z3495* is required for O157 LEE expression in order to promote adherence to host cells. Meanwhile, we performed FAS assays, which showed that the percentage of AE lesion formation on HeLa cells in Δ*z3495* significantly decreased compared with that on O157 WT (300 cells per strain, shown in Figure 2E,F). Meanwhile, the average pedestal number in Δ*z3495*-infected cells was lower (3.78) than that of the O157 WT (9.40, Figure 2G). Complementation of *z3495* restored the virulence capacity of Δ*z3495* to the wild-type level (Figure 2E–G), indicating that *z3495* is required for O157 adherence to host cells and LEE gene expression to promote its pathogenicity. 

### 3.3. Z3495 Regulates LEE Expression Indirectly

**Figure 3:**
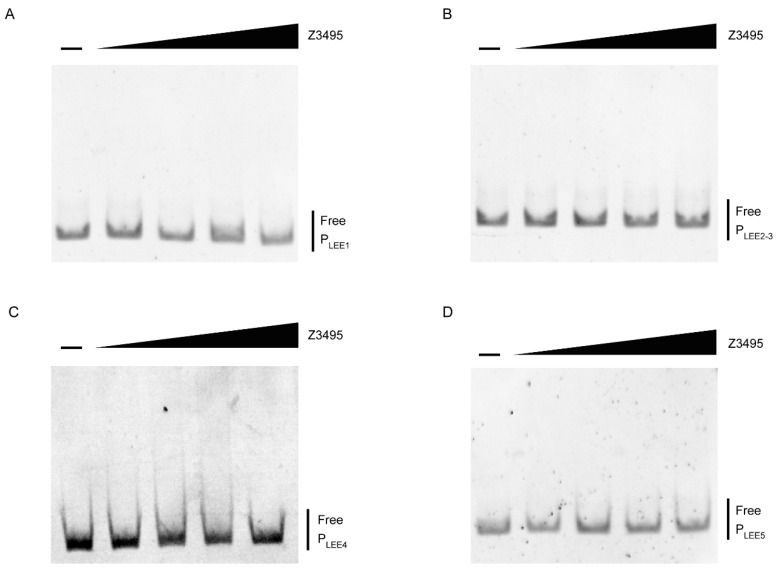
Z3495 regulates LEE expression indirectly. (**A**–**D**) EMSA of the specific binding of Z3495 to P_LEE1_ (**A**), P_LEE2/3_ (**B**), P_LEE4_ (**C**), and P_LEE5_ (**D**).

The LEE island is organized into five polycistronic operons (LEE1–5). The first gene of the LEE1 operon, *ler*, is the master transcriptional regulator. To investigate whether *z3495* regulates LEE genes directly, we performed electrophoretic mobility shift assays (EMSAs) to evaluate the binding of *z3495* to LEE promoters (PLEE1, PLEE2/3, PLEE4, and PLEE5). MBP-tagged Z3495 protein was purified in *E. coli* strain BL21 (DE3). The promoter of LEE1-5 and *rpoS* fragment was amplified by PCR using genomic DNA from EHEC O157:H7 strain EDL933. The results showed that there were no noticeable band shifts observed for the LEE promoter (Figure 3A–D), which indicated that *z3495* regulated the expression of LEE genes indirectly. 

### 3.4. Z3495 Contributes to O157 Adherence and LEE Expression via rcsF

**Figure 4:**
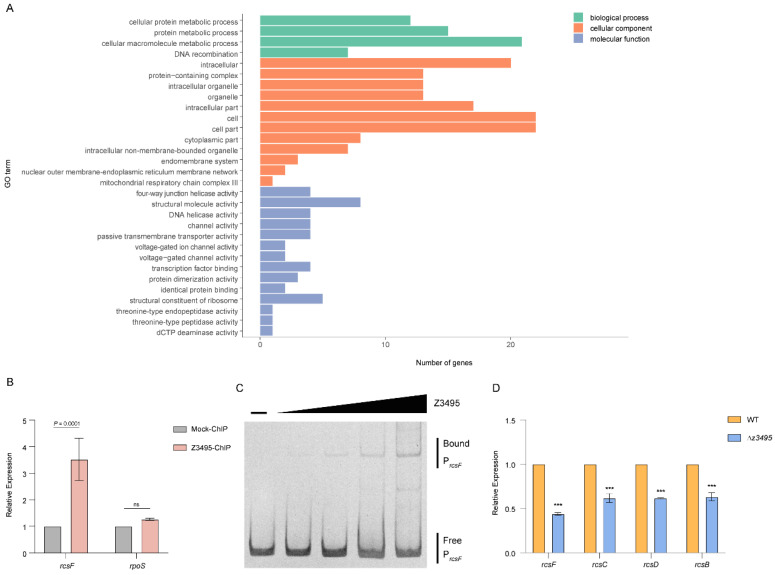
Z3495 binds to the promoter of *rcsF*. (**A**) GO enrichment analysis of enriched loci in Z3495-ChIP samples. (**B**) The fold enrichment of the promoters for *rcsF* and the negative control (*rpoS*) in the chromatin immunoprecipitation assay. (**C**) EMSA of the specific binding of Z3495 to the promoter of *rcsF*. (**D**) qRT-PCR of the expression of Rcs system genes in WT and Δ*z3495*. Data represent means ± SD (n = 3). *** *p* ≤ 0.001 (Student’s *t*-test), ns, not significant.

The above results showed that Z3495 regulates LEE expression indirectly. To investigate the mechanism of LEE expression regulated by *z3495* more comprehensively, chromatin immunoprecipitation sequencing (ChIP-Seq) was performed to investigate which Z3495 regulates LEE gene expression was performed. Accordingly, 3 × FLAG-tagged full-length *z3495* was overexpressed from plasmid pTrc99A and transformed into a *z3495* mutant. Chromatin bound to Z3495 was crosslinked, sheared, purified, and sequenced. Sequence reads were obtained from ChIP-seq assays using a FLAG-specific antibody and mapped to the EHEC O157:H7 genome. A total of 97 enriched loci containing specific Z3495-binding peaks were identified with significant enrichments compared with the control sample (*p*-value of e^−5^, Supplementary Table S4). We categorized the biological processes of Z3495 targets based on gene ontology (GO) [27]. The loci were significantly enriched in the cellular protein metabolic process and cellular macromolecule metabolic process in the biological process; intracellular part and cell part in the cellular component; and transcription factor binding and structural molecule activity in molecular function. More detailed GO enrichment analysis results are shown in Figure 4A. 

In the ChIP-seq results, we identified that a Z3495-binding peak located in the promoter region of *rcsF*, which was characterized by contributing to O157 virulence, was enriched in the Z3495-ChIP samples but was absent in the control samples. To verify this result, ChIP-qPCR was performed. Consistent with the ChIP-seq results, the ChIP-qPCR results showed that the promoter of *rcsF* was enriched 3.52-fold in the Z3495-ChIP samples compared with the mock ChIP samples (Figure 4B). In contrast, the fold enrichment of *rpoS* showed no significant differences between the Z3495-ChIP and mock ChIP samples (Figure 4B), indicating that Z3495 specifically binds to the promoter of *rcsF*.

To further prove that *z3495* directly regulates *rscF* expression by binding to its promoter, an EMSA assay was performed using purified MBP-tagged *z3495*. The results showed that with increasing concentrations of Z3495 protein, migrating bands were observed for the promoter of *rcsF* (Figure 4C). By contrast, under the same conditions, when we used a DNA fragment derived from the *rpoS* gene as the negative control, Z3495 binding was eliminated (Figure S6). These results indicated that Z3495 can bind to the *rcsF* promoter *in vitro*. To exclude the effect of MBP, we also transformed the pMal-c5X vector into *E. coli* strain BL21 (DE3) and purified the MBP protein to detect its ability to bind to the *rcsF* promoter. The result showed that MBP did not bind to the *rcsF* promoter (Figure S7), which proved that the binding of MBP-tagged *z3495* to the *rcsF* promoter is not caused by MBP. 

Previous studies demonstrated that RcsF contributes to the function of RcsC/RcsD/RcsB phosphorelay to promote O157 virulence by increasing LEE gene transcription [28]. Therefore, we performed a qRT-PCR assay to detect the expression of the Rcs system in Δ*z3495* to investigate whether the expression of the Rcs system is regulated by *z3495*. qRT-PCR analysis revealed that the expression of *rcsF*, *rcsB*, *rcsC*, and *rcsD* were reduced in Δ*z3495* compared with the WT strain (Figure 4D), indicating that the expression of the Rcs system was positively regulated by *z3495* via binding to the promoter of *rcsF* directly. In conclusion, these results demonstrated that *z3495* regulates the expression of *rscF* by binding to its promoter, which further regulates the expression of the Rcs system, contributing to O157 virulence. 

### 3.5. Z3495 Is a Widespread Regulator of Virulence in Pathogenic Bacteria

**Figure 5:**
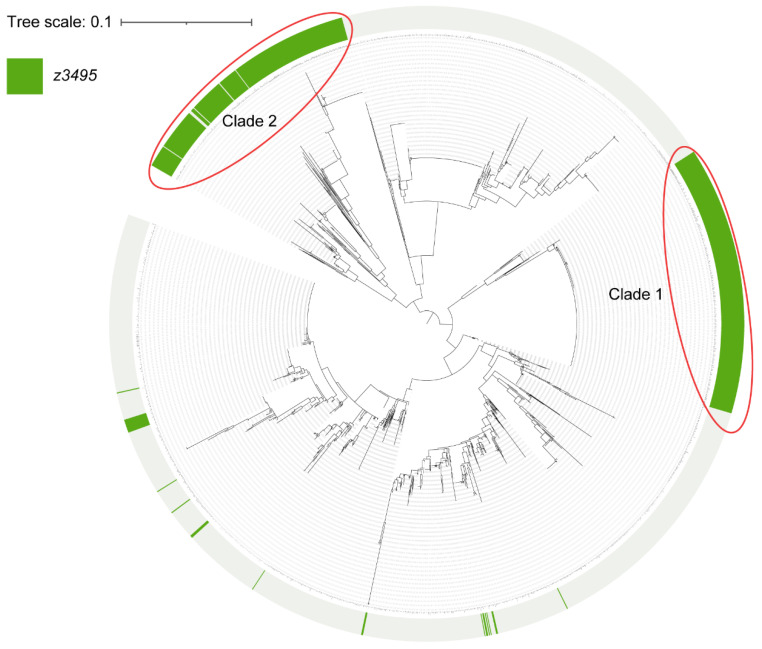
Phylogenetic analysis of 2134 publicly available *E. coli* complete genomes. The presence of *z3495* is indicated by the green semicircle on the outer ring.

To investigate the distribution of *z3495* in *E. coli*, a comparative genomics analysis was performed using all 2134 publicly available *E. coli* complete genomes. Phylogenetic analysis revealed that *z3495* is highly conserved and predominantly distributed in two distinct clades. Clade 1 includes EHEC O157:H7 and enteropathogenic *E. coli* strain O55:H7. Clade 2 mainly comprises extraintestinal pathogenic *E. coli* strains, such as ST2747, ST648, and uropathogenic *E. coli* MS6198 (Figure 5). This result indicated that Z3495 was independently acquired via lateral gene transfer events. BLASTP searches against the NCBI nonredundant protein database revealed that Z3495 orthologs also existed in Citrobacter, Salmonella, and Klebsiella, suggesting that *z3495* is widely distributed in pathogenic bacteria.

## 4. Discussion

By acquiring virulence factors, harmonizing commensals can become niche-specific pathogens that have a competitive advantage over the resident microbiota [29]. There are 177 O islands in the genome of EHEC O157:H7 strain EDL933, which do not exist in nonpathogenic *E. coli* K-12 strain MG1655 [13]. EHEC usually colonizes the large intestine, causing severe diseases. The pathogenicity of EHEC O157:H7 is mainly conferred by the large pathogenicity island, OI-148, termed LEE, which contains a type III secretion system (T3SS), and its effectors, OI-45 and OI-93, harbor the *Stx1* and *Stx2* genes which encode the subunits of Shiga toxin [15]. In addition to OI-148, OI-45, and OI-93, a growing number of OI-associated genes have been assigned a function related to O157 virulence, such as OI-9, OI-19, OI-26, OI-36, OI-50, OI-57, OI-71, OI-79, and OI-122 [15,25,30]. There are, however, many OIs that remain uncharacterized in terms of their functions and evolutionary histories. Here, we found that OI-97 is required for bacterial adherence to host cells, LEE gene expression, and colonization in the mouse intestine, contributing to the virulence of O157. Our study further proves the importance of OIs in the pathogenicity of EHEC and enhances our understanding of the functions of OIs. 

LEE transcriptional regulation is extremely complex, involving at least three regulator classes: LEE encoded, global, and horizontally transferred [11,30,31]. These LEE regulators ensure LEE expression only under optimal environmental conditions while preventing expression to avoid the intense metabolic costs and ensuring survival in other environments [32]. In this study, we identified that a virulence regulatory protein, Z3495, encoded in OI-97, induces the expression of *rcsF*, contributing to the pathogenicity of EHEC O157:H7 by promoting the expression of LEE. Z3495 sequence conservation and its presence in different *E. coli* strain pathotypes indicate that the acquisition of this gene is important for pathogenic bacteria evolution. Our study significantly enhances our understanding of bacterial virulence control and reveals the increased complexity of the regulatory network of LEE genes.

Z3495 belongs to the group of LysR-type transcriptional regulators (LTTRs), the most abundant type of transcriptional regulator, whose members have a conserved structure with an N-terminal DNA-binding helix–turn–helix motif and a C-terminal co-inducer-binding domain [33,34,35]. LTTRs regulate a diverse set of genes in *E. coli*, *Salmonella enterica* serovar Typhimurium, and *Yersinia enterocolitica*, that are involved in virulence, metabolism, quorum sensing, and motility [36,37,38,39,40]. More than 44 LTTRs that regulate genes associated with bacterial stress response and systemic virulence have been documented in the *Salmonella* genome [41]. Here, we found that Z3495 plays an important role in O157 virulence, significantly expanding our insight into the regulatory function and scope of LTTRs.

In conclusion, we identified a transcriptional regulator that activates the expression of *ler*, Z3495, contributing to EHEC O157:H7 virulence. This factor was required for the induction of LEE genes during initial host adherence and colonization in the large intestine in vivo. It provides a potential target for the development of new therapeutics for the EHEC O157:H7 infection.

## Data Availability

The ChIP-seq data available at Sequence Read Archive database (SRA: PRJNA1045878); All data available in the manuscript and the Supplementary Materials.

## Supplementary Materials

The following supporting information can be downloaded at: https://www.mdpi.com/article/10.3390/microorganisms12010140/s1.
