# Supplementary material for: Z3495, a LysR-Type Transcriptional Regulator Encoded in O Island 97, Regulates Virulence Gene Expression in Enterohemorrhagic Escherichia coli O157:H7
Source: Microorganisms. 2024 Jan 10;12(1):140. doi: 10.3390/microorganisms12010140 (PMC10819331; doi:10.3390/microorganisms12010140)
Supplement: Supplementary file 1 [file microorganisms-12-00140-s001.zip › Supplementary Figure.pdf]

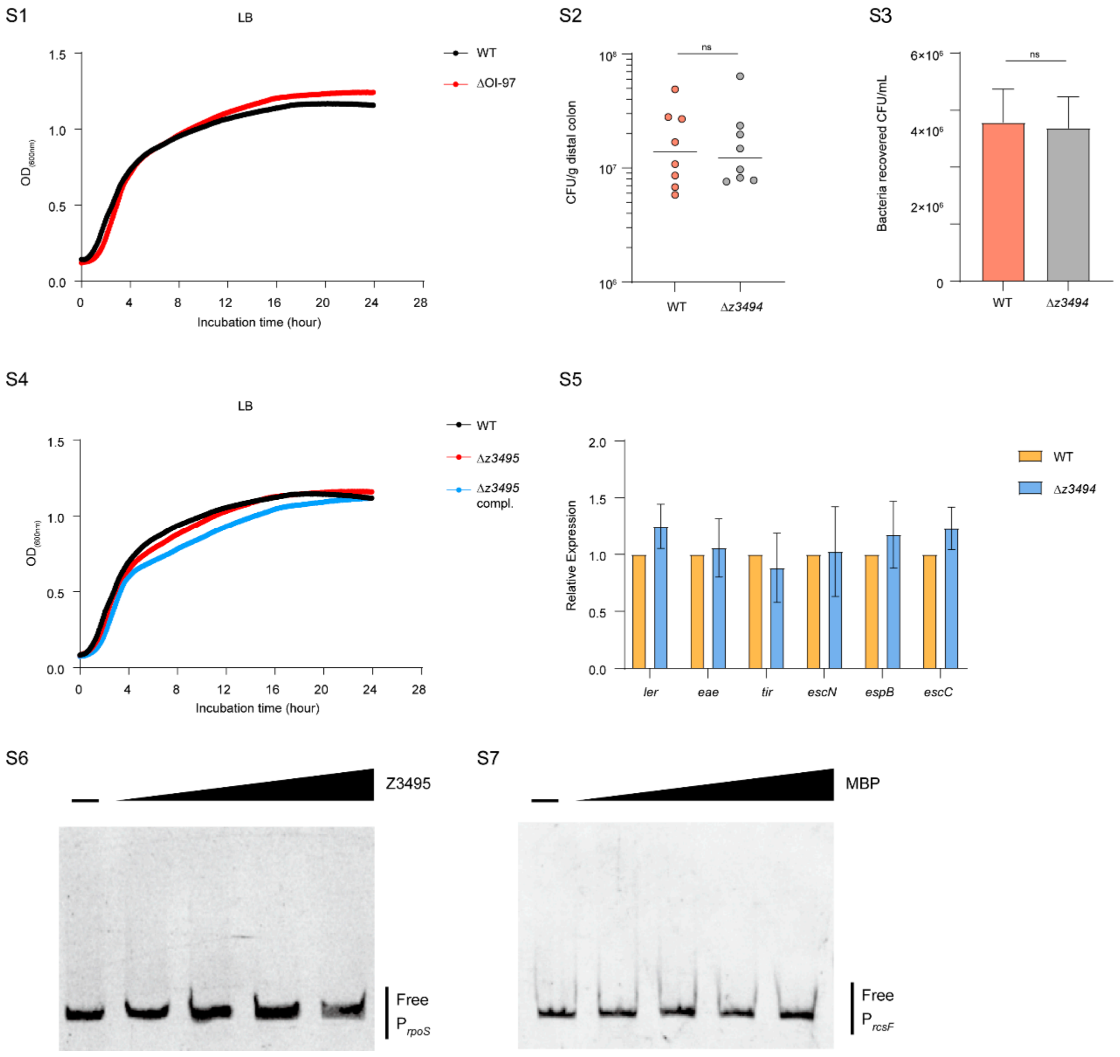

Figure S1. Growth curves of NMEC and ΔOI-97 in LB.

Figure S2. Evaluation of the adherence capacity of O157 WT and Δ*z3494* in the distal colon of mice at 6 h.

Figure S3. Adhesion of O157 WT and Δ*z3494* strains to HeLa cells in DMEM.

Figure S4. Growth curves of NMEC, Δ*z3495* and Δ*z3495* complement strain in LB

Figure S5. qRT-PCR of the expression of LEE genes in WT and Δ*z3494*.

Figure S6. EMSA of the specific binding of Z3495 to *rpoS*.

Figure S7. EMSA of the specific binding of MBP to *rcsF*.

Data represent mean ± SD (n = 3).
